# Anti-glomerular basement membrane disease in children: a brief overview

**DOI:** 10.1007/s00467-021-05333-z

**Published:** 2021-11-12

**Authors:** Thomas Dowsett, Louise Oni

**Affiliations:** 1 Department of Paediatric Nephrology, Royal Manchester Children’s Hospital, Oxford Road, Manchester, M13 9WL UK; 2 Department of Paediatric Nephrology, Alder Hey Children’s NHS Foundation Trust Hospital, Eaton Road, Liverpool, L12 2AP UK; 3 Department of Women’s and Children’s Health, Institute of Life Course and Medical Sciences, University of Liverpool, Eaton Road, Liverpool, L12 2AP UK

**Keywords:** Anti-GBM, Glomerulonephritis, Children

## Abstract

Anti-glomerular basement membrane disease (Anti-GBM), previously known as Goodpasture syndrome, is an extremely rare cause of rapidly progressive glomerulonephritis and chronic kidney disease stage 5 (CKD5) in children. It is associated with acute pulmonary haemorrhage and it has a poor prognosis. It is classified as an autoimmune, small-vessel vasculitis caused by autoantibody formation against the alpha-3 chain in type IV collagen found in the glomerular basement membrane. Evidence of anti-GBM antibodies in serum or histologically are required for diagnosis. Treatment in children is based on very limited adult data and often involves the use of acute apheresis to rapidly remove circulating factors coupled with intensive immunosuppression such as cyclophosphamide and intravenous corticosteroids. There is also an emerging role for the use of biologic agents such as B cell depletion. The evidence base in children with anti-GBM disease is extremely limited. Multi-centre international collaboration is required to provide insight into this disease, better describe its prognosis and work towards improving outcomes. This review article summarises the key features of this disease in children, highlights treatment options and considers areas of unmet need.

## Introduction

Anti-glomerular basement membrane disease (Anti-GBM), previously known as Goodpasture’s disease or syndrome, is an extremely rare cause of glomerulonephritis (GN) and chronic kidney disease stage 5 (CKD 5) in children. The literature relating to anti-GBM disease in children is limited to a small number of case reports and retrospective case series. Although rare, it is characterised by rapidly progressive glomerulonephritis (RPGN) and it is associated with a poor prognosis [1]. This concise review summarises the key features of this disease, discusses management, including the role of apheresis therapy, and considers areas of unmet need to help improve future outcomes in children.

## Incidence and clinical features

Anti-GBM disease is extremely uncommon in children; however, it is responsible for ~ 20% of all causes of RPGN. Defining its precise incidence in children remains a challenge. In adults it has an incidence of 0.5–1.0 cases per million population per year [2]. In adults, it shows a bimodal distribution with peaks in the 3^rd^ and 6^th^ decades of life [3]. The US data determined that anti-GBM disease accounted for 0.4% (24/6,560 cases) of all paediatric CKD 5 [4]. Other studies have reported that anti-GBM disease accounts for 3% of crescenteric GN in children [5]. The disease prevalence demonstrates some seasonal variation and geographical clustering which may be due to infectious triggers that include upper and lower respiratory tract infections such as influenza A and more recently the severe acute respiratory syndrome coronavirus-2 (SARS-COV-2) evident during the COVID-19 pandemic [6, 7]. The sparse literature suggests a 2:1 predominance in females in children. In adults, it appears to be more common in males [8].

The disease classically presents with rapidly progressive GN in 80–90% of cases necessitating acute kidney replacement therapy and this presentation is similar between adults and children with the disease [6]. Most patients report a degree of prodromal illness including lethargy and malaise in the weeks prior to presentation. Up to 60% of cases will also develop pulmonary haemorrhage while a minority may present with pulmonary involvement in isolation [6, 8]. Pulmonary involvement can vary significantly, from life-threatening haemoptysis to asymptomatic radiographic or bronchoscopy findings alone [8, 9]. Pulmonary symptoms commonly include shortness of breath, wheeze, haemoptysis and chest pain. Signs and symptoms of kidney involvement are those typical of an acute GN including severe hypertension and fluid overload. Haematuria may be either microscopic or macroscopic. Cerebral involvement due to primary cerebral small vessel angiitis is reported and usually presents with seizures [8, 10]. In adults, exposure to cigarette smoking and hydrocarbons is a risk factor for developing the disease, but this has not been reflected in paediatric case reports and may be due to the low likelihood of exposure to these triggers in children [11].

Detection of anti-GBM antibodies, either in serum or histologically, assist in formulating the diagnosis [3]. In approximately 10% of patients with anti-GBM disease circulating antibodies would not be detected. This may be due to either false negative results within the enzyme immunoassays or due to genuine absence of circulating antibodies, and therefore, histological evidence of disease, through lung or kidney tissue, is important in cases where there remains a high clinical suspicion of disease [12]. It is the antibody deposition that distinguishes anti-GBM disease from other types of glomerulo-nephritides such as post-infectious, immune-complex and isolated antinuclear cytoplasmic antibody (ANCA)-associated GN. Kidney histology classically identifies extensive crescent formation affecting > 80% of the glomeruli on light microscopy and immunofluorescence (IF) detects linear IgG deposition along the GBM. In some cases with severe, extensive glomerular inflammation, the histological features may be distorted and challenging to accurately report. Additionally, the linear IgG deposition may also be seen in other inflammatory diseases including diabetes, paraproteins, lupus nephritis or fibrillary GN. Genetic studies have shown an association between Human Leucocyte Antigens (HLA) DRB1*1051 and DRB1*1502 while HLA-DR7 and DR1 appear to be somewhat protective [3].

## Disease pathophysiology

**Fig. 1:**
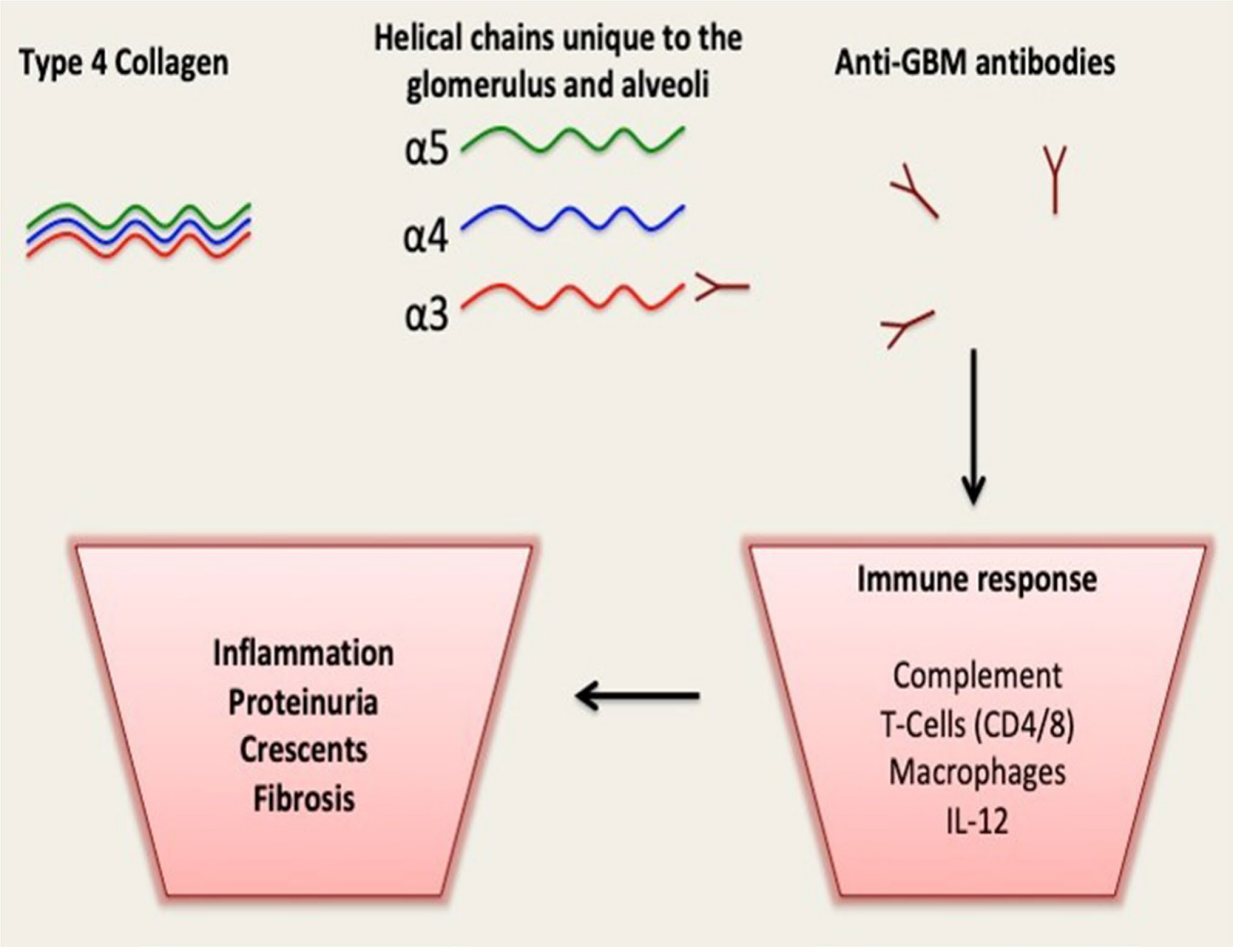
The α 3, 4, 5 chains that are specific to type IV collagen found in the basement membranes of the glomerulus and alveoli. Environmental exposure to certain risk factors reveal hidden antigenic epitopes on the α 3 chain leading to the autoimmune production of anti-GBM antibodies and the subsequent inflammatory response

The glomerular basement membrane is a critical part of the glomerular filtration barrier. Type IV collagen is a major contributor to maintaining the stability of the GBM [13]. Anti-GBM disease is classified as an autoimmune, small-vessel vasculitis caused by pathogenic autoantibody formation usually of the IgG class, with IgG1 and IgG3 predominating, that target the alpha-3 chain in type IV collagen found in the GBM (Fig. 1) [3]. This collagen chain subtype is present in both alveolar and kidney basement membranes explaining the disease phenotype. Binding of IgG antibodies to the GBM lead to classical complement activation and a neutrophilic inflammatory response [3]. The epitope responsible for antibody binding is hidden within the protein hexamer of type IV collagen and two conformational epitopes have been defined at different residues within the alpha-3-NC1 domain that are believed to correlate with outcomes (reported as type EA and EB) [14]. It is proposed that an immunological or environmental exposure, such as infections, cigarette smoking or hydrocarbons, uncover the NC1 domain that then acts as an antigen to the immune system [15]. The innate immune system also contributes to the inflammatory process of this disease with T cells contributing to direct cell mediated glomerular injury and T regulatory cells proposed to have the ability to suppress aspects of the autoimmune and alloimmune response [16].

## Overlap with other conditions

ANCA-associated vasculitis (AAV) and anti-GBM are included in the spectrum of pulmonary-renal syndromes as they may present with a similar combination of kidney and lung manifestations [17]. Cases of dual ANCA and anti-GBM antibody positivity are widely reported (17% of reported cases in children) and it has been suggested that the initial presence of ANCA may be the trigger responsible for exposing the epitope in the alpha-3 chain [8]. Dual positivity is associated with a poorer prognosis, and thus, early, aggressive treatment is recommended [18]. Patients may also present with features overlapping with membranous nephropathy at a higher rate than would be expected by chance and atypical presentations of this disease are reported [19].

In patients with Alport syndrome, genetic mutations coding for the type IV collagen chains are responsible for progressive kidney disease [6]. Cases of allo-immune production of anti-GBM antibodies (de-novo anti-GBM) have occurred following kidney transplantation in patients with Alport syndrome [6]. It is important to note that this occurs more frequently in those with gene deletions as opposed to point mutations. Importantly, antibodies develop against the alpha-5 chain in type IV collagen (in contrast to the alpha-3 chain in autoimmune anti-GBM disease) meaning standard assays may not detect development of anti-GBM antibodies in this cohort [20]. Studies have reported up to 14% of kidney transplant patients in this group show the histological changes consistent with anti-GBM disease. However, only a small number of these had clinical features of anti-GBM GN post transplantation (1.9%) [21]. If clinical recurrence of anti-GBM disease does occur in transplanted patients, it is reported to occur early and can lead to rapid graft loss [20].

## Management

The management of anti-GBM disease in children is based on the approach derived from the management in adult disease and clearly described within the recent KDIGO glomerular disease guidelines (2021) [22]. Principles include removal of the pathogenic circulating antibody and potential immune mediators with acute apheresis; ceasing production of antibodies with cyclophosphamide and/or B cell depleting agents; and reducing inflammation with corticosteroids and/or adjunctive immune modifying agents such as mycophenolate mofetil (MMF) [23]. Supportive therapy includes the use of kidney replacement therapy, usually haemodialysis and antihypertensive agents when needed. There are no literature reports using acute peritoneal dialysis in this condition probably due to the need for secure vascular access to permit apheresis therapy.

### Apheresis therapy

Anti-GBM is a recognised indication for the use of apheresis treatment in cases presenting with acute GN not requiring dialysis who have diffuse alveolar haemorrhage, or dialysis dependent acute GN without diffuse alveolar haemorrhage [24]. Even in cases where irreversible crescenteric CKD 5 is present, aggressive treatment should still be initiated if there is evidence of diffuse alveolar haemorrhage due to its associated risk with mortality (100% mortality from historical data) [23]. Guidelines suggest a 1–1.5 plasma volume exchange using either fresh frozen plasma or 5% albumin depending on the risk of bleeding. Daily or alternate day treatment with at least 14 sessions or until antibodies are no longer detectable are generally recommended [25]. As children are typically also treated with aggressive immunosuppression, it is difficult to reliably report on the effectiveness of apheresis on kidney outcomes. A review of the apheresis protocols used within the literature in children with anti-GBM is summarised in Table 1 [8, 26–32]. Immunoadsorption leading to more specific removal of pathogenic immunoglobulins has been reported [33]. A comparison study ‘of immunoadsorption’ in adults failed to demonstrate any additional benefit over double filtration plasmapheresis [34].

**Table 1:** A summary of published case reports and case series describing children receiving apheresis therapy for anti-GBM disease in the literature during the past 20 years

Author	Year	Number of patients	Age, years (median (IQR) for cohort data)	Significant comorbidities	Dialysis	Immunosuppressive treatment	Apheresis type	Number of sessions	Outcome
Hagan	2015	1	7	Nephrectomy for xanthogranulomatous pyelonephritis	HD	MP, cyclophosphamide	Single volume TPE	19 sessions	Partial response, CKD stage 4 at 12 months
Maxted	2020	5	14 (2–19)	NS	HD	MP (*n* = 5), cyclophosphamide (*n* = 3), IV cyclophosphamide and MMF (*n* = 1)	DFPP	Range 3–10 sessions	Complete response (*n* = 3), partial response (*n* = 2) with CKD
Jiao	2012	1	15	Turner syndrome	HD	MP, cyclophosphamide	Single volume TPE	9 sessions	No kidney response, improvement in pulmonary haemorrhage
Weiss	2012	22	13 (9–16)	NS	NS	NS	Single volume TPE	Median 5 (IQR 3–7) sessions	NS
Helander	2021	1	2	ANCA positive antibodies	HD	MP, cyclophosphamide, rituximab	Single volume TPE	15 sessions	No kidney response, improvement in pulmonary haemorrhage
Mannemud dhu	2019	1	15	Common variable immunodeficiency	HD	MP, cyclophosphamide, rituximab	Single volume TPE	10 sessions	No kidney response
Bayat	2012	1	14	Tetralogy of Fallot, cigarette smoker	Not required	MP, cyclophosphamide	Single volume TPE	4 sessions	Complete response
Mayer	2020	1	17	NS	HD	MP, cyclophosphamide, rituximab	Single volume TPE	9 sessions	No kidney response
Agarwal	2017	1	11	ANCA positive antibodies	HD	MP, cyclophosphamide	Single volume TPE	22 sessions	Partial response, ‘near normal’ kidney function

Abbreviations: *HD* haemodialysis, *NS* not specified, *MP* methylprednisolone, *IV* intravenous, *MMF* mycophenolate mofetil, *TPE* therapeutic plasma exchange, *DFPP* double filtration plasmapheresis, *eGFR* estimated glomerular filtration rate

### Immunosuppressive therapy

First-line immunosuppressive therapy in this disease is cyclophosphamide and high dose corticosteroids as recommended by the KDIGO guidelines for the management of GN [23]. Cyclophosphamide has recognised toxic side effects that include gonadal toxicity that seems to be influenced by the total cumulative dose received and the stage of pubertal development in the child. Pre-pubertal children are at lowest risk of future gonadal failure with rates reported to be < 10% in pre-pubertal boys receiving < 400 mg/kg total dose of cyclophosphamide [35]. Rituximab, a B-cell depleting agent, is advised if cyclophosphamide is contra-indicated and it could be considered as a disease adjunct in severe cases. Small case series in adults demonstrated measurable improvement in respiratory disease using rituximab as an induction agent, but they failed to demonstrate improvements in kidney outcomes [36]. MMF has been used in a small number of patients with reported success for disease induction and maintenance [37].

## Prognosis

Unlike many other autoimmune conditions, anti-GBM disease does not tend to run a relapsing, remitting course. The initial presentation is therefore usually responsible for disease-associated morbidity and mortality. In a large series of adult patients (*n* = 119), a third of patients required intensive care unit admission on first presentation, 78% needed kidney replacement therapy (KRT) and 82% received plasma exchange therapy. The 1-year survival rate was 95% and the 3-year survival rate was 92% [38]. In this series, the use of plasma exchange was associated with better survival. Regarding morbidity, kidney failure is the largest consequence of this disease with rates > 46% in adult patients and the risk of kidney failure is associated with increased creatinine at presentation, need for KRT, less cumulative dose of cyclophosphamide, and histological features that include extra capillary proliferation, capsular rupture, interstitial fibrosis and hyaline thrombi [38]. The literature surrounding the prognosis in children is very limited. In one case series (US) of four paediatric patients, one died at presentation from pulmonary haemorrhage and three patients received the treatment course outlined above. Of these patients, one recovered kidney function at 1 year and two required ongoing kidney replacement therapy and eventual transplantation, suggesting a similar 50% rate of kidney failure in children [11]. KDIGO recommends a period of 6 months with undetectable levels of anti-GBM antibody prior to considering transplantation [23]. Graft loss and mortality post-transplant are reported to be similar for anti-GBM disease when compared to other immune-mediated diseases and disease recurrence following transplantation is rare but recognised in 2–4% of patients [39, 40].

## Areas of unmet need

The evidence base for the diagnosis and management of anti-GBM disease in children is derived from adult data. The proposed standard treatments are based on adult studies and seem to be well accepted [22]. Further research is needed to identify treatment protocols that would improve current standard therapy in this rare condition. These studies will need to involve adult patients due to the rarity of the disease in children. Improved understanding of the immune mechanisms involved in this disease will identify novel targets for treatment, such as specific biologic inhibitors of the immune system or exploring the role of the T cell functions, particularly T regulatory cells that may help to suppress disease. Pre-clinical studies have demonstrated promise using Fostamatinib treatment, an inhibitor of the tyrosine kinase signalling pathway, in its ability to interfere with crescent formation. National and international collaboration to prospectively report cases would identify associations in the development of the disease, define current practice and provide more up to date outcomes of the current treatment protocols used in children.

## Summary

Anti-GBM disease is a rare entity in childhood but it is associated with a significant level of morbidity and acute mortality. The autoimmune development of antibodies towards type IV collagen in the glomerular and alveolar basement membranes leads to patients typically presenting with RPGN and pulmonary haemorrhage. Evidence of anti-GBM antibodies in serum or histologically is required for diagnosis. Treatment in children is based on adult data and involves the use of apheresis and intensive, strong immunosuppression such as cyclophosphamide. Multi-centre international collaboration is required to provide insight into this disease and to improve its management in children.
